# Analysis of the mutational landscape of classic Hodgkin lymphoma identifies disease heterogeneity and potential therapeutic targets

**DOI:** 10.18632/oncotarget.22799

**Published:** 2017-11-30

**Authors:** Elena Mata, Antonio Díaz-López, Ana M. Martín-Moreno, Margarita Sánchez-Beato, Ignacio Varela, María J. Mestre, Carlos Santonja, Fernando Burgos, Javier Menárguez, Mónica Estévez, Mariano Provencio, Beatriz Sánchez-Espiridión, Eva Díaz, Carlos Montalbán, Miguel A. Piris, Juan F. García

**Affiliations:** ^1^ Department of Pathology and Translational Research, MD Anderson Cancer Center Madrid, Madrid, Spain; ^2^ Lymphoma Research Group, Medical Oncology Department, Instituto Investigación Sanitaria Puerta de Hierro (IDIPHIM), Madrid, Spain; ^3^ Instituto de Biomedicina y Biotecnología de Cantabria, IBBTEC (CSIC, Universidad de Cantabria), Santander, Spain; ^4^ Department of Pathology, Hospital Universitario de Móstoles, Madrid, Spain; ^5^ Department of Pathology, Fundación Jiménez Díaz, Madrid, Spain; ^6^ Department of Pathology, Hospital Severo Ochoa, Madrid, Spain; ^7^ Department of Pathology, Hospital General Universitario Gregorio Marañón, Madrid, Spain; ^8^ Department of Hematology, MD Anderson Cancer Center Madrid, Madrid, Spain; ^9^ Department of Molecular Translational Pathology, The University of Texas MD Anderson Cancer Center, Houston, Texas, USA

**Keywords:** mutational analysis, Hodgkin lymphoma, B-cell receptor, BTK, therapeutic target

## Abstract

Defining the mutational landscape of classic Hodgkin lymphoma is still a major research goal. New targeted next-generation sequencing (NGS) techniques may identify pathogenic mechanisms and new therapeutic opportunities related to this disease. We describe the mutational profile of a series of 57 cHL cases, enriched in Hodgkin and Reed-Sternberg (HRS) cells.

Overall, the results confirm the presence of strong genomic heterogeneity. However, several variants were consistently detected in genes related to relevant signaling pathways, such as GM-CSF/IL-3, CBP/EP300, JAK/STAT, NF-kappaB, and numerous variants of genes affecting the B-cell receptor (BCR) pathway, such as *BTK*, *CARD11*, *BCL10*, among others. This unexpectedly high prevalence of mutations affecting the BCR pathway suggests some requirement for active BCR signaling for cHL cell viability. Additionally, incubation of a panel of cHL cellular models with selective BTK inhibitors *in vitro* constrains cell proliferation and causes cell death. Our results indicate new pathogenic mechanisms and therapeutic opportunities in this disease.

## INTRODUCTION

Classic Hodgkin lymphoma (cHL) consists of a clonal proliferation of the distinctive Hodgkin and Reed Sternberg (HRS) cells, diluted in a reactive inflammatory microenvironment [1]. HRS cells have customarily been characterized by a defective B cell expression program [2], probably as a result of the downregulated expression of numerous B cell transcription factors [3], and epigenetic mechanisms [4]. Thus, although HRS cells are derived from mature B cells, they have largely lost the B cell phenotype and are unable to express immunoglobulins / B-cell receptor (BCR) and other B cell surface markers.

Few studies have attempted to identify gene mutations in cHL, and only mutations in specific members of the NF-kappaB and JAK/STAT pathways have been consistently identified [5–8]. A major limitation of gene sequencing of cHL is the scarcity of tumoral HRS cells, which account for fewer than 1-2% of all cells, as identified by standard CD30 immunostaining.

The advances in next-generation sequencing (NGS) technologies have allowed rapid genome-wide characterization of single-nucleotide variants (SNVs) and indels in cancer, and the genetic landscapes of many solid tumors and hematological malignancies have been demonstrated [9–12]. The first whole exome sequencing analyses of primary HRS cells have recently been described, in which beta-2-microglobulin (*B2M*) was identified as the most commonly altered gene [12]. However, this study focused on the detection of highly prevalent mutations, and had obvious limitations for the detection of subclonal variants due to the shallow depth of sequencing inherent in full exome analysis technologies.

In recent years, the sensitivity and specificity of NGS techniques have been greatly improved by simultaneously testing selected genes, arranged in specific comprehensive gene panels. This approach is also useful when sample material is limited, and even for formalin-fixed paraffin-embedded (FFPE) tissue samples. We hypothesized that combining sequencing techniques with increased coverage, and more sensitive and specific bioinformatic algorithms, could also allow identification of pathogenic SNVs that are present at low frequencies in this disease.

Here we analyze the genomic heterogeneity of a large series of 57 cHL cases. We used a semiconductor-based (Ion Torrent) sequencing platform with a custom-designed gene panel coupled with HRS cell-enrichment procedures to establish the mutational landscape of this tumor in greater detail. Several variants were consistently identified in genes related to relevant signaling pathways, such as GM-CSF/IL-3, JAK/STAT, NF-kappaB, and also many variants of genes affecting the BCR pathway, such as *BTK*, *CARD11*, *BCL10*, and others. Moreover, selective-BTK inhibitors were found to constrain cell proliferation and cause cell death in cHL cellular models with comparable efficiencies to other cell B-cell lymphoma models.

## RESULTS

### Recurrent mutations in cHL

Clinical characteristics of the patients are described in Table 1. It is of particular note that 34% of cases in this series had primary refractory cHL, and 28.3% of tumors were EBV-positive.

**Table 1:** Clinical characteristics of patients

FEATURE	N	%
**Age (years)**		
< 45	37	68.52
≥ 45	17	31.48
**Gender**		
Male	26	48.15
Female	28	51.85
**IPS**		
0-2	35	64.81
≥ 3	19	35.19
**Outcome**		
Refractory	18	33.96
Complete response	35	66.04
**Ann Arbor Stage**		
< IV	36	67.92
≥ IV	17	32.08
**Histology**		
Nodular sclerosis	34	59.64
Mixed cellularity	13	22.8
Lymphocyte-rich	6	10.53
NA	4	7.03
**EBV**		
Positive	15	28.3
Negative	38	71.7

Using the described NGS targeted protocol, the number of candidate somatic SNVs detected per case was found to range from 27 to 587. Cases with low numbers of SNVs detected in each duplicate had high concordance ratios (Supplementary Figure 1), whereas cases with high numbers of SNVs showed low concordance, which means that there is either very extensive clonal diversity (many mutations with very low variant allele frequencies) or that some SNVs actually represent sequencing errors in cases with poorer DNA preservation. This observation confirms the presence of strong genomic heterogeneity, and reinforces the relevance of filtering SNVs using duplicate analyses in this study.

**Figure 1:**
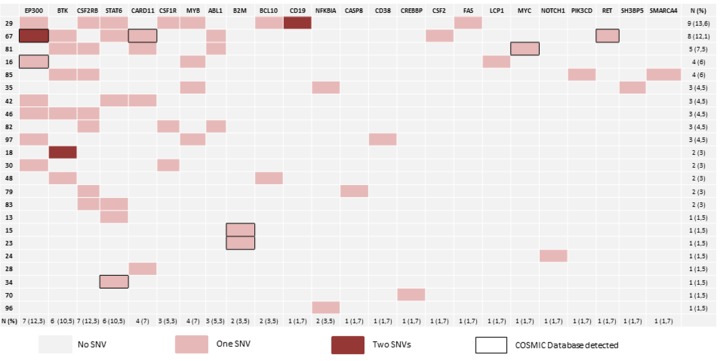
Mutational landscape The diagram shows the distribution of gene variants. 40.3% of samples have at least 1 SNV after filtering. We identified variants in 66.6% of the selected genes. One SNV is indicated in red, while genes with more than two SNVs are indicated in dark red. The final two columns indicate the number and percentage of mutated genes per case. The bottom two rows indicate the number and percentage of cases with at least one SNV.

Figure 1 and Supplementary Table 4 summarize the details of the SNVs. After filtering, we found 63 concordant SNVs in 57 samples. The mean read depth of the targeted regions was 740.32. Non-synonymous SNVs were identified in 23 out of the 57 cases (40.35%). We found a high percentage of C>T changes (nearly 46%), probably due to the dipyrimidine context (Supplementary Table 4) [13]. As other authors have noted [8], some of the cases had more than one SNV per gene, as in the cases of *EP300*, *BTK*, *CSF1R* and *CD19*.

We found the predicted damaging effect of the SNVs in 24 out of the 36 genes analyzed (66.6%). Of these, 4 genes had mutation rates greater than 10%: *CSF2RB*, *EP300*, *STAT6*, and *BTK* (Figure 1 and Supplementary Figure 2). This finding may reflect the importance of the deregulation of relevant signaling pathways (such as JAK/STAT and BTK), and the relevance of epigenetic deregulation.

**Figure 2:**
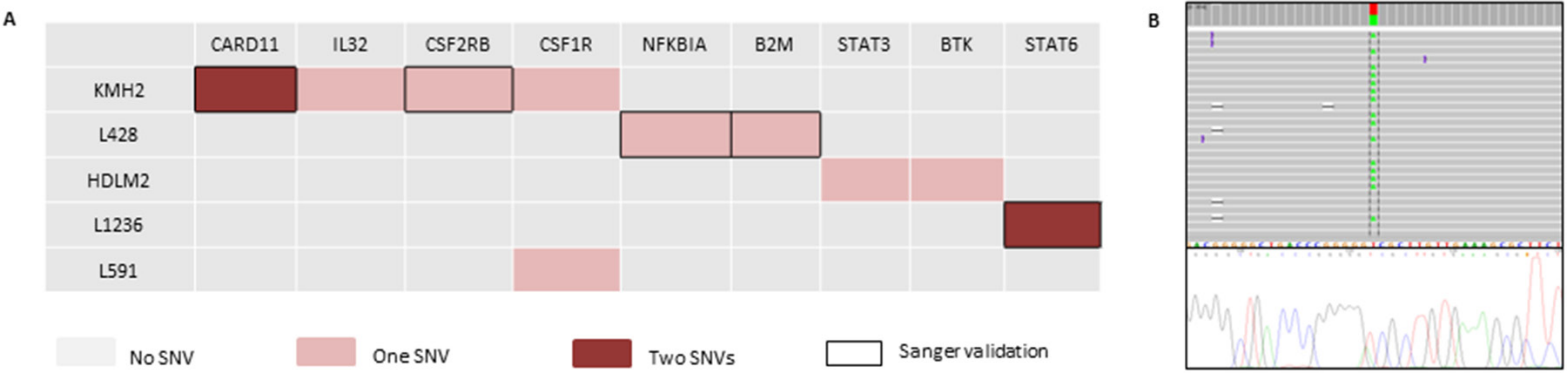
Mutational landscape in 6 cHL-derived cell lines and Sanger sequencing validation **(A)** The diagram shows the distribution of mutations per cell line. One SNV is indicated in red, while two SNVs per gene are indicated in dark red. Black boxes correspond to SNVs validated by Sanger sequencing. **(B)** Representative example, CARD11 mutation in the KMH2 cell line; IGV diagram (Ion Torrent sequencing) and Sanger sequencing representation.

Some of the variants were also identified in cHL-derived cell lines, and validated using Sanger sequencing. They affected genes such as *CARD11, STAT6, CSF2RB, B2M*, and *NFKBIA* (Figure 2 and Supplementary Table 5). Consistent with our results, some of these SNVs, such as those of *STAT6*, *B2M*, and *NFKBIA*, have been described as mutations in previous NGS analyses of cHL cell lines [14].

### Functional studies

**Figure 3:**
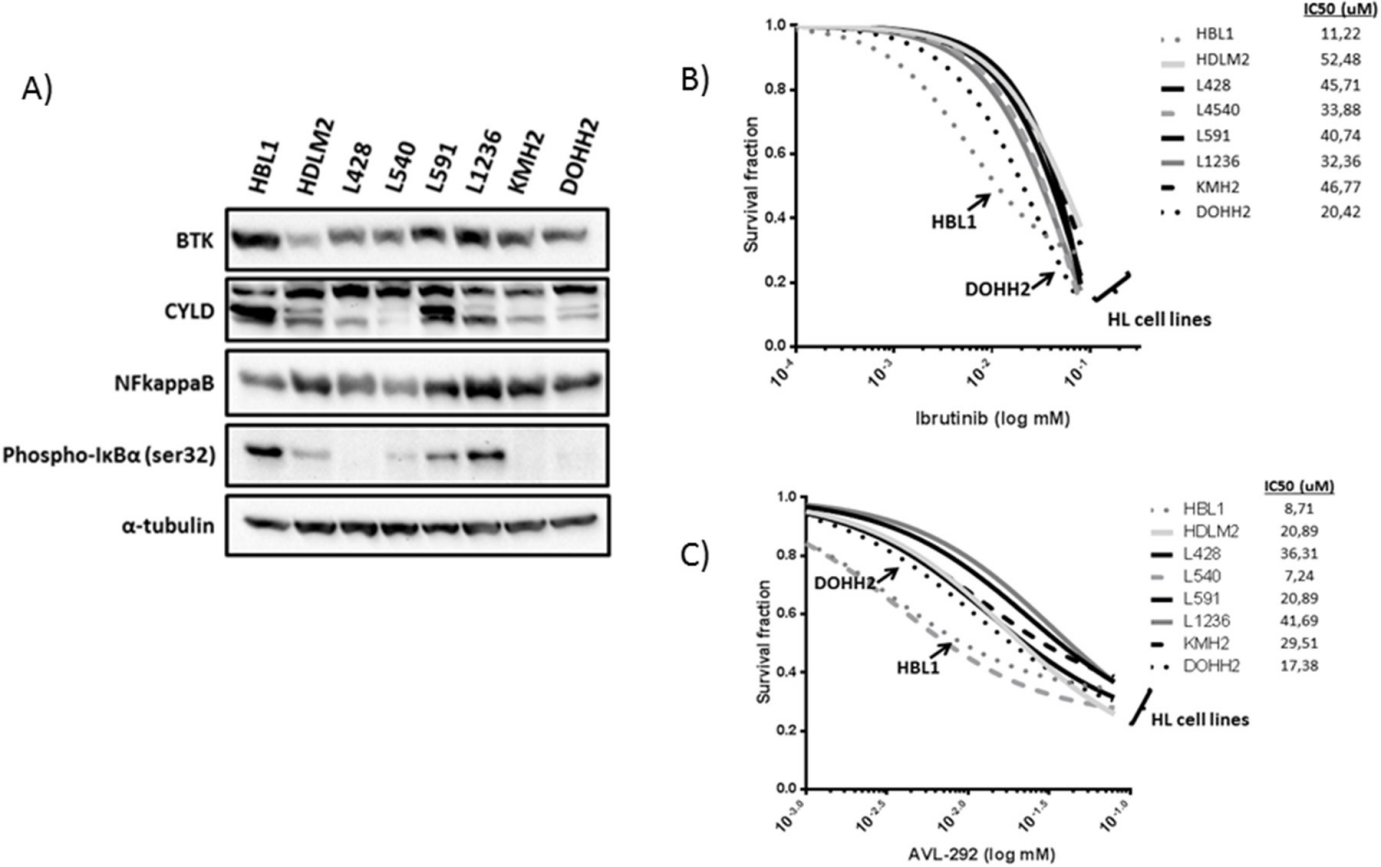
Functional studies in cHL-derived and DLBCL-derived cell lines **(A)** Western blot analysis of total BTK, CYLD truncation, NFKB (p52 isoform), and phospho-IkBα. **(B** and **C)** Activity of BTK selective inhibitors in cHL-derived and DLBCL-derived cell lines. IC_50_ range calculation of HBL1, HDLM2, L540, and L1236 cell lines in the presence of different concentrations of Ibrutinib or AVL-292, after 48 hours.

Unexpectedly, we identified a large number of mutations affecting the *BTK* gene (10.3% of cases) and other members of the BCR pathway (such as *CARD11*, *BCL10*, and *NFKBIA*, which were present in up to 21% of cases), suggesting that there are some dependencies of active BCR signaling despite the known absence of BCR expression in HRS cells. To evaluate BCR signaling activation in cHL, 6 cHL cell lines, the HBL1 ABC-type DLBCL cell line, the DOHH2 GC-type DLBCL cell line, and the control HEK293T and HeLa cell lines were interrogated. The NFkappaB subunit p52 was strongly expressed in all the cHLs analyzed and both DLBCL cell lines, providing further evidence of the well-known role of NFkappaB in lymphocyte survival (Figure 3A). Since NFkappaB activation may be the result of activation of several survival pathways, we analyzed the expression of other proteins involved in BCR signaling. BTK protein expression was found in all cHL cell lines at similar levels, unlike BTK Tyr223 phosphorylation, which was only found in DLBCL cells (HBL1 and DOHH2) and some of the cHL cell lines (L428 and HDML2) (Figure 3A). In addition to BTK expression, activation of NFkappaB via BCR signaling was explored by CYLD cleavage, a direct downstream target protein of the BCR pathway. CYLD cleavage by western blot was observed in all cHL cell lines and DLBCL cell lines at similar levels, supporting the hypothesis that there is some level of basal activation of the BCR cascade in cHL.

We therefore examined the activity of selective BTK inhibitors in cHL cell lines. First, we used ibrutinib, a specific BTK inhibitor approved for therapy for various B cell-derived malignancies. Ibrutinib toxicity curves showed a similar IC_50_ range of 10-50 μM in both models: DLBCL (HBL1) and cHL cell lines, without significant differences (Figure 3B). To confirm these findings we incubated the cells with a second experimental BTK inhibitor (AVL-292), which yielded similar IC_50_ ranges in all the cell lines analyzed. Indeed, the IC_50_ after BTK inhibition with ibrutinib was similar in other cell models of B-cell malignancy, such as mantle cell lymphoma (data not shown).

**Figure 4:**
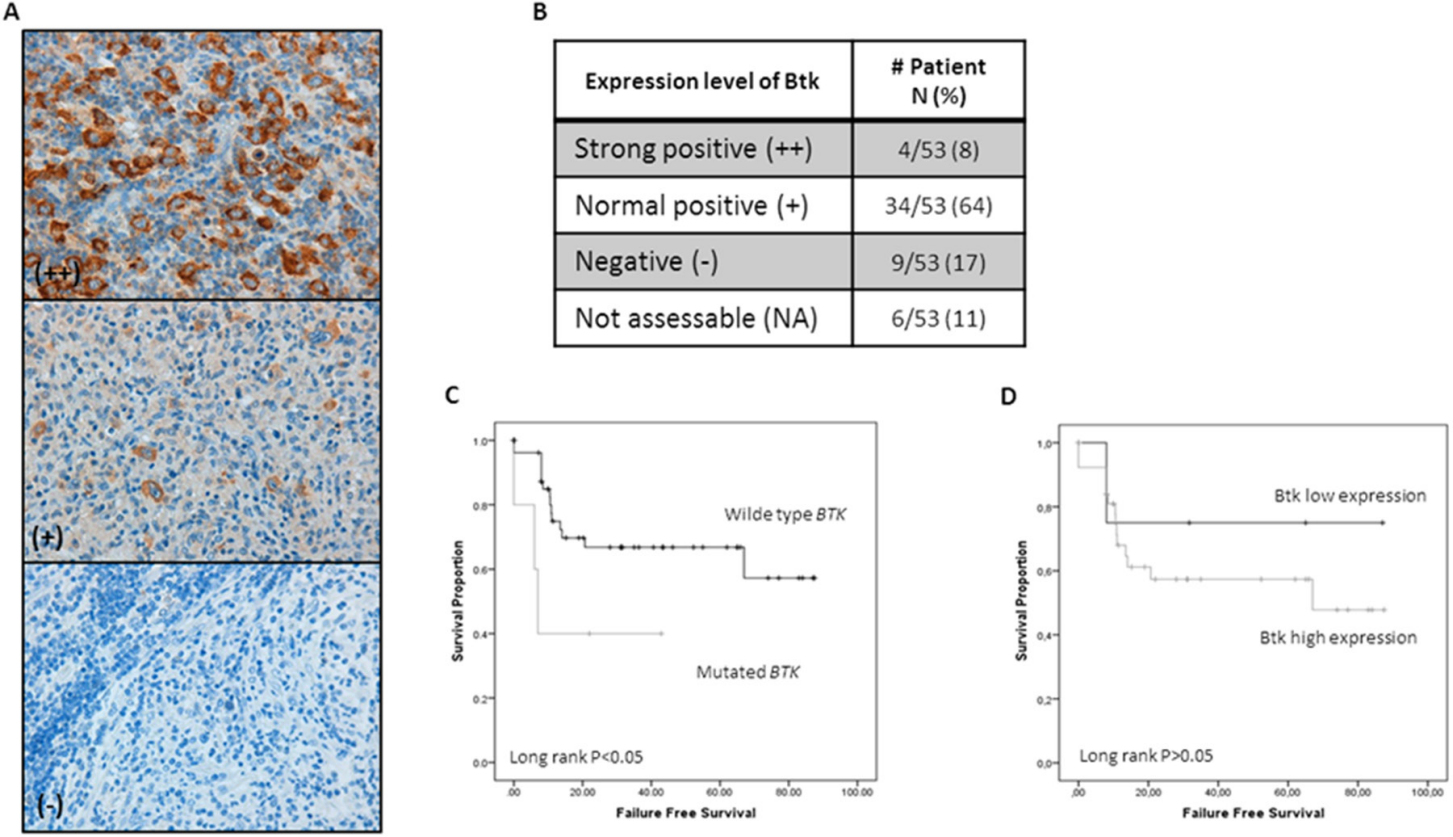
Btk IHC expression in HRS cells and Its correlation with survival **(A)** Representative examples of IHC for Btk expression in cHL tissues. **(B)** Distribution of Btk protein expression in our series. Positivity (+) was concluded for cases with a level of expression comparable to that seen in normal germinal center B lymphocytes. **(C)** Kaplan–Meier survival curves demonstrate longer FFS in wt-BTK cases (P<0.05). **(D)** Survival curves demonstrate a longer FFS in cases with a low level of expression of Btk protein (P=n.s.).

Consistent with this, IHC staining of the tumor samples using TMAs showed common BTK protein expression in the neoplastic HRS cells (72% of cases), with unusually strong expression in 8% of cases (Figure 4). We also found a significant correlation between FFS and the presence of *BTK* gene mutations (Figure 4C) and a just-insignificant correlation with BTK protein expression (Figure 4D).

## DISCUSSION

Although cHL is generally a highly curable disease, a significant fraction of patients nevertheless have relapsing disease and eventually die due to treatment resistance or late treatment-associated toxicities [15]. Thus, the identification of new therapeutic alternatives and the better biological characterization of the subgroup of patients with refractory disease remain major research goals. Here we describe the mutational profile of cHL using data initially generated in the initial discovery cohort and extend these observations to a large series of 57 cHLs using targeted deep sequencing with a restricted list of 36 genes and duplicate experiments.

We detected recurrent mutations in 24 genes. Other authors have reported some of the genes commonly mutated in cHL, such as *NFKBIA* [16] and *FAS* [17]. It is of note that many of the variants identified here have also been detected in the recent NGS analysis of primary HRS cells [12], including *ABL1*, *B2M*, *CARD11*, *CSF2RB*, *MYB*, *NFKB2*, *NFKBIA*, and *STAT6*, among others. Differences in isolation techniques, sequencing methods, filtering criteria, and limitations due to small sample sizes may be responsible for some of the discrepancies in frequencies. We also identified some previously unreported SNVs in *BTK* and *EP300*.

We identified several SNVs in genes that had previously been described in NGS studies of DLBCL. We consistently found variants affecting *CARD11*, *STAT6*, *CREBBP*, and *CMYB*, as described in ABC-type DLBCL [9, 11], and *STAT6*, as known in primary mediastinal large B-cell lymphoma [10]. These findings are consistent with the well-known molecular similarities between cHL and these lymphoma types [10, 18].

The most frequent genetic lesions potentially affect relevant signaling pathways in B cell biology (see Supplementary Materials): JAK/STAT, NF-kappaB, and numerous variants of genes affecting the B cell receptor (BCR) pathway, such as *BTK*, *CARD11*, and *BCL10*, among others. Epigenetic regulation also seems to be a common target for mutational events (*EP300*, *CREBBP*).

*EP300* encodes an adenovirus E1A-associated cellular p300 transcriptional coactivator protein and functions as a histone acetyltransferase and regulates transcription via chromatin remodeling. Histone acetylation gives an epigenetic tag for transcriptional activation. It mediates cAMP-gene regulation by binding specifically to phosphorylated CREB protein. It also functions as an acetyltransferase for non-histone targets. Mutations in the histone acetylation domain of EP300 are present in 14% of the samples, similar to what is seen in DLBCL and follicular lymphoma [19, 20]. Interestingly, the inhibition of the CBP/p300 bromodomain has been recently introduced as a promising therapy for lymphoproliferative disorders [21].

*CSF2RB* (colony stimulating factor 2 receptor beta common subunit) encodes the common beta chain of the high-affinity receptor for interleukin-3, interleukin-5, and granulocyte-macrophage colony-stimulating factor. Its function is critical for the activation of the JAK/STAT and MAP kinase pathways. In this series, we found low allele frequency SNVs in 7 samples (12.3%). One particular SNV, V212I, is recurrent in 4 of the cHL cases. This alteration is located in the fibronectin type III domain (FIB). The RGD sequence (Arginine-Glycine-Aspartic acid), which is involved in the interaction with integrin, is located in this domain, and modulates a variety of cell adhesion events associated with thrombosis, inflammation and tumor metastasis [22].

*STAT6* (Signal Transducer and Activator of Transcription 6, Interleukin-4 Induced) encodes a member of the STAT family of transcription factors, plays a central role in exerting IL4-mediated biological responses, and induces the expression of BCL2L1/BCL-X(L), which is responsible for the anti-apoptotic activity of IL4. We found 7 SNVs in 7 cases (12.3% of samples), most of which are located in the DNA binding domain, as has been found in previous studies of primary mediastinal DLBCL. Functional studies in cell lines have shown that mutations in this domain reduced DNA-binding ability [10].

*BTK* is a non-receptor tyrosine kinase that is indispensable for B lymphocyte development, differentiation, and signaling. Binding of antigens to the BCR triggers signaling that ultimately leads to B-cell activation. This gene has been implicated in the pathogenesis of chronic lymphocytic leukemia, mantle cell lymphoma, DLBCL and other B-cell malignancies. We have seen 7 different SNVs in 6 samples (10.3% of our series). Ibrutinib is an irreversible inhibitor of BTK, binding to cysteine 481 in the protein tyrosine kinase domain (ATP binding site). Woyach et al [23] have recently identified a cysteine-to-serine mutation at the ibrutinib binding site in treated patients that results in a protein that is only reversibly inhibited, and could be associated with therapy resistance. This specific alteration is different from all the SNVs that we found in our series, located at different functional domains, mainly affecting the PH domain, which is involved in establishing the location of the protein and its membrane anchoring. The BTK subcellular localization in tumor cells is a key process for the activation of the pathway and *BTK* mutations affecting this functional domain may also be related with BCR signaling activation [24].

Unexpectedly, we observed numerous variants of genes affecting the BCR pathway, such as *BTK*, *CARD11*, and *BCL10*. These findings suggest that there may be a requirement for active BCR signaling to ensure cHL cell viability, even though BCR is not expressed by HRS cells. Consistent with this interpretation, we found evidence of the basal activation of the pathway in cHL cell lines, and that the incubation of a panel of *in vitro* cell models with specific BTK inhibitors constrains cell proliferation and causes cell death. However, from our results we must also recognize the potential overlap between BCR signaling and the canonical NF-kappaB pathway, making it difficult to establish firm conclusions. In addition, some BTK-independent effect of the inhibitors in different cell models cannot be ruled out. Previous studies reported BTK expression in only approximately 20% of patients with cHL [25], but here we detected very frequent BTK protein expression in cHL tumors, as well as some correlation between protein expression, *BTK* gene mutation and survival. In accordance with our observations, the clinical value of ibrutinib in cHL patients was partially demonstrated in a recent communication by Hamadani et al [26], who administered ibrutinib as a single agent to two heavily pretreated patients with primary refractory cHL. One of the patients showed near-complete regression of disease two months after the initiation of therapy that lasted for 4 months. The second patient showed a stable complete response 4 months after the initiation of ibrutinib.

In conclusion, our results confirm the presence of strong genomic heterogeneity in cHL. However, it appears that recurrent targets are subject to functional mutations and suggest the existence of some tumor dependencies that could be exploited for therapeutic purposes. The activity of selective BTK inhibitors in patients with refractory cHL, as well as the inhibition of the CBP/p300 bromodomain and JAK/STAT pathways, need to be evaluated in prospective clinical studies.

## MATERIALS AND METHODS

### Patient samples

Pretreatment FFPE tumor samples and clinical data from 57 cHL patients were obtained from the files of participant institutions. cHL was confirmed histologically in all cases by central review.

Samples were collected in accordance with the technical and ethical procedures of the Spanish National Biobank Network, including anonymization processes and written informed consent, according to the Helsinki Declaration. Approval was obtained from the institutional review board (Clinical Research Ethical Committee, ref. 354/12).

### Gene selection and Ampliseq design

Target genes were selected by analyzing the mutational profile of cHL with Illumina GAII and Sure Select technology (Agilent Technologies, Santa Clara, CA, USA) in an initial discovery cohort of 7 tumor tissue samples (freshly frozen) from 4 cHL cases, with a targeted analysis of 522 genes involved in lymphomagenesis and B and T cell-related pathways (Supplementary Table 1). Sequences were analyzed with the RAMSES (Realignment-Assisted Minimum Evidence Spotter) algorithm [27], which has been specifically designed to detect low-frequency variants for the Illumina platform. This software uses minimum high confidence evidence, based on the use of multiple short read aligners, of the presence of sequence variants at a specific position. Considering the great extent of coverage obtained in this project, we established a minimum of 20 high-confidence reads reporting a different base in a specific mutation to conclude the presence of a mutation. We also filtered out all the variants reported in dbSNP and the 1000 Genomes project as probable germline variants. Small insertions and deletions (indels) were identified using PINDEL [28].

Two predictive algorithms (PROVEAN [29] and Alamut [13]) indicated that 54 variants of 48 genes had a deleterious effect on protein function. From these results, we selected 25 genes filtered by established criteria based on sequencing quality and discovery cohort population frequency (consistently mutated in at least two samples). As additional controls, we added 6 genes (detected in single samples) to this design with previously reported mutations in diffuse large B-cell lymphomas (DLBCLs) or in cHL, as well as 4 genes of biological relevance in this type of lymphoma (Supplementary Table 2). These 35 coding regions from target genes were included in an Ampliseq Custom Panel for sequencing using the Ion Torrent platform.

### Sequencing techniques

To isolate DNA, tumor samples were enriched in HRS cells by selecting the most representative regions (those containing more than 10% of tumor cells), using a 1-mm-diameter puncher (Quick-Ray Tissue Microarray System, IHC World, Woodstock, MD, USA) from tumor issues of at least 1-mm thickness.

DNA was extracted from FFPE tissue and from cell lines using standard procedures. Ion Torrent adapter-ligated libraries were constructed using an Ampliseq Custom Panel (Thermo Fisher Scientific, NY, USA), including the 35 selected genes distributed in 353 amplicons, starting with 10 ng of genome DNA and following the manufacturer´s protocol.

Sample emulsion PCRs and enrichment were performed using Ion PGM Template OT2 200 Kit (Thermo Fisher Scientific), and the libraries were sequenced using 318 v2 chips with Ion PGM Sequencing 200 v2 (Thermo Fisher Scientific). To minimize false-positive rates, all sequencing procedures were done in duplicate (parallel libraries constructed from two independent PCRs from separate aliquots of isolated genomic DNA). For filtering polymorphisms and germinal variants we included the available germline DNA from 5 cases in order to generate control pool DNA.

### Variant calling and screening for somatic mutations

The sequencing data were analyzed with the Torrent Suite program with the “variant caller” plug-in program, selecting somatic low stringency mode to detect SNVs with low allele frequency. We restricted the analyses to variants with a total coverage of at least 100x, and a minimum variant coverage of 10x. In addition to filtering with the control pool of germline DNAs, we also filtered variants with an allele frequency >40%, to minimize the detection of germline variants. To eliminate erroneous base calling, all variants were examined with Integrative Genomics Viewer (IGV) software [30], comparing each duplicated sample and discarding non-concordant variants. In addition, to evaluate the reproducibility of this approach, we calculated the concordance ratios between duplicates (Supplementary Figure 1) [31]. Finally, functional consequences of the SNVs were predicted using the PROVEAN and Alamut algorithms, filtering variants with no damaging effect in the prediction [13, 29].

We also analyzed six cHL-derived cell lines (L428, L1236, HDLM2, L540, KMH2, and L591), using the same sequencing procedure and filtering criteria, and by conventional Sanger sequencing to validate high allele frequency mutations (Supplementary Table 3). Cell lines were newly obtained from the German Collection of Microorganisms and Cell Cultures (DSMZ, Braunschweigh, Germany). All cell lines were authenticated by DMSZ using DNA profiling

### Cell culture, drug treatment, protein extraction, and western blot analyses

For *in vitro* studies we included the six cHL cell lines and DLBCL cell lines (HBL1 and DOHH2) (DSMZ).

For functional experiments, we purchased ibrutinib (Selleckchem, Houston, TX) and AVL-292 (MedchemExpress, Monmouth Junction, NJ) selective BTK (Bruton tyrosine kinase) inhibitors. To calculate the half-maximal inhibitory concentration (IC_50_), 20,000 cells were incubated with various concentrations of ibrutinib or AVL-292 for 48 hours. Viable cells were quantified by the AlamarBlue protocol (Fisher Scientific, Waltham, MA), following the manufacturer’s instructions. Experiments were performed in duplicate and repeated at least three times.

Apoptosis was estimated after 8 hours of treatment at 37°C, by analyzing PARP cleavage by western blot. Additional western blot analyses were done using monoclonal antibodies against CYLD N-terminal (ab153698) (ABCAM, Cambridge, UK), Phospho-BTK (Tyr223), Phospho-IκBα (Ser32) (14D4) (Cell Signaling, Danvers, MA), BTK (7F12H4) (Santa Cruz Biotechnology, Santa Cruz, CA), NFκB p52 (MERCK, Madrid, Spain) and α-tubulin (Sigma Aldrich, St. Louis, MO).

### Immunohistochemistry (IHC)

Representative tumor areas from FFPE tumor samples were included in duplicate in a tissue microarray (TMA). IHC analysis was performed using heat-induced epitope retrieval and standard procedures, using anti-Btk monoclonal antibody (7F12H4, Santa Cruz). Protein expression was quantified using an automated scan, Chroma Vision Systems-ACIS III (DAKO, Glostrup, Denmark), as previously described [32].

### Statistical analyses

Correlations between variables and overall survival (OS) and failure-free survival (FFS) were estimated by the Kaplan–Meier method and the curves were compared using the log-rank test. Differences were considered statistically significant for values of P<0.05. All statistical analyses were performed using SPSS version 17.0 (SPSS Inc., Chicago, IL).

## SUPPLEMENTARY MATERIALS FIGURES AND TABLES










