# Supplementary material for: Analysis of the mutational landscape of classic Hodgkin lymphoma identifies disease heterogeneity and potential therapeutic targets
Source: Oncotarget. 2017 Nov 30;8(67):111386–95. doi: 10.18632/oncotarget.22799 (PMC5762329; doi:10.18632/oncotarget.22799)
Supplement: Supplementary file 1 [file oncotarget-08-111386-s001.pdf]

Analysis of the mutational landscape of classic Hodgkin lymphoma identifies disease heterogeneity and potential therapeutic targets

SUPPLEMENTARY MATERIALS

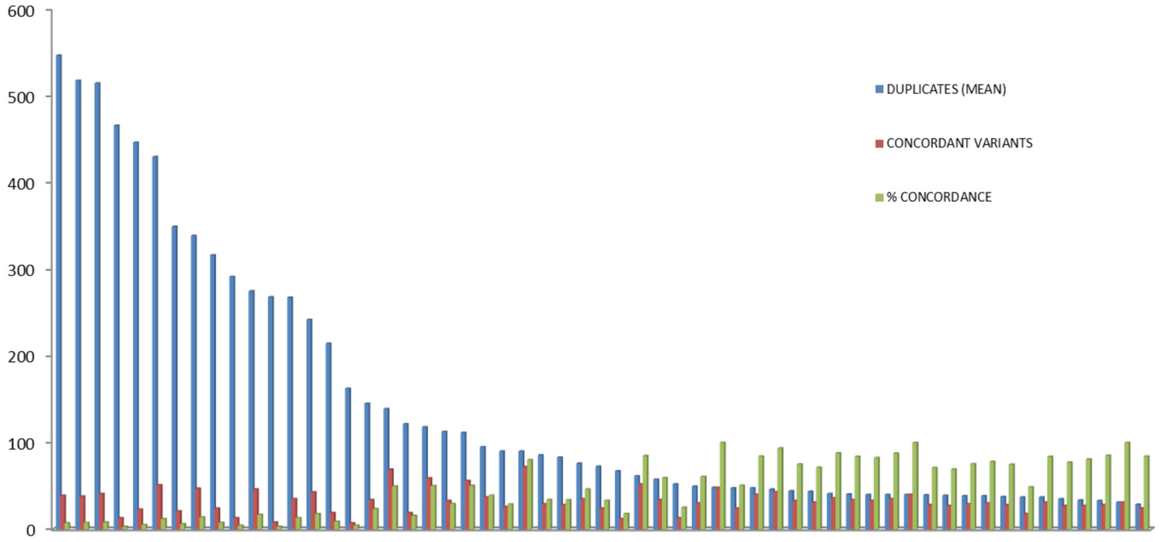

Supplementary Figure 1: Concordance ratios of SNVs detected in the samples. The figure shows the differences between the number of SNVs detected in each case (27-587), and the percentage concordance between duplicates (range 3-100%). The mean read depth of the targeted exonic regions was 927.5 and the mean and SD in the cases of high allele frequency were 16.34 and 23.40, respectively.

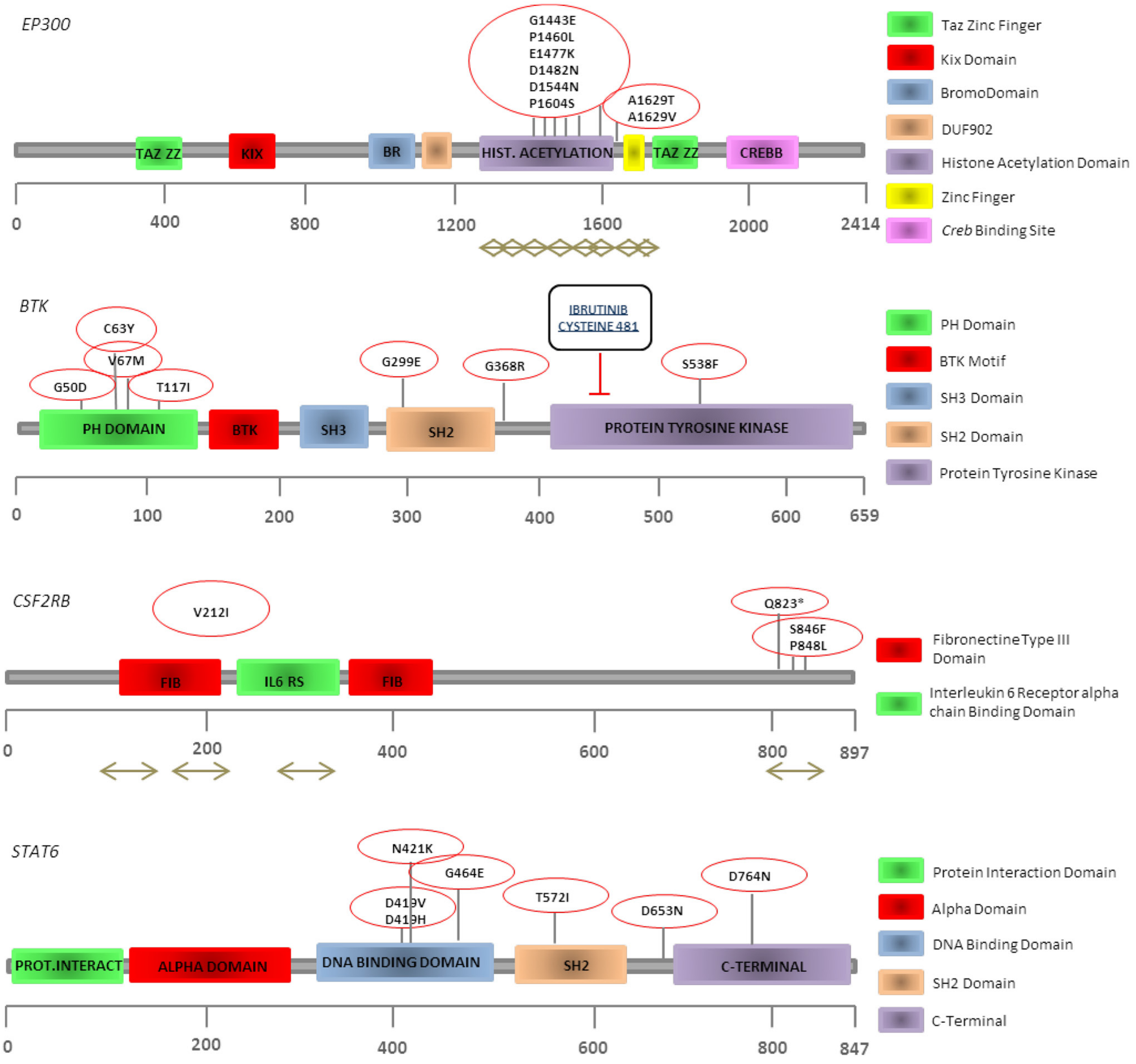

Supplementary Figure 2: Recurrent mutations in cHL. Schematic representation of alterations in CSF2RB, EP300, STAT6, and BTK genes (genes mutated in at least 10% of tumor samples). These data may reflect the importance of the deregulation of relevant signaling pathways (such as JAK/STAT and BTK), and the relevance of epigenetic deregulation. In BTK, ibrutinib binds cysteine 481 at the ATP binding site, which inhibits its phosphorylation and abolishes downstream BCR signaling. The brown double-headed arrow represents the sequenced regions of each gene; in *STAT6* and *BTK* the coverage was at least 98%.

Supplementary Table 1: Target selection design (Sure Select)

See Supplementary File 1

Supplementary Table 2: Ampliseq custom panel

This design consists of 353 amplicons distributed in 35 genes. Each amplicon is around 150 bp long (optimized for FFPE), with 97% mean coverage in the coding regions. Selection criteria were established on the basis of various characteristics: 25 genes were selected from the previous results using the Illumina platform for NGS (selection of genes or regions consistently mutated in at least two samples); 7 additional genes were selected on the basis of previous reports of being frequently mutated in cHL and/or their frequent mutations in DLBCL (in particular, primary mediastinal LBCL); and 3 genes were included in the panel because of their biological relevance to cHL biology.

See Supplementary File 2

Supplementary Table 3: Selected genes, primers and PCR conditions for Sanger sequencing of DNA extracted from cell lines

See Supplementary File 3

Supplementary Table 4: SNV summary

After filtering, we found 63 SNVs in 57 samples. The mean read depth of the targeted exonic regions was 740.35 and the mean and SD in cases of high allele frequency were 5.82 and 6.37, respectively. Non-synonymous SNVs were identified in 23 out of 57 cases (40.35%) and in 24 out of 36 genes (66.6%). We found a high percentage of C>T changes (nearly 47%), probably due to the dipyrimidine context.

See Supplementary File 4

Supplementary Table 5: Summary of SNVs found in cell lines

cHL cell line	Chromosome	Chromosomal position	Base change	Allele frequency (%)	Coverage	Gene ID	Type	Amino acid change
KMH2	chr7	2978436	GTCTGA>-	51.0	4935	CARD11	Deletion	DS296
KMH2	chr7	2956982	T>A	45.3	1029	CARD11	SNV	D882V
KMH2	chr16	3117390	G>A	25.2	5328	IL32	SNV	D10N
KMH2	chr22	37325765	G>A	48.4	3123	CSF2RB	SNV	V212I
KMH2	chr5	149450132	T>C	46.5	5884	CSF1R	SNV	H362R
KMH2	chr14	35871136	TTC>-	92.8	318	NFKBIA	Deletion	--
HDLM2	chr17	40474420	C>A	50.0	814	STAT3	SNV	D661Y
HDLM2	chrX	100611835	A>G	17.9	280	BTK	SNV	I429T
L428	chr14	35871707	G>A	100.0	2653	NFKBIA	SNV	Q267*
L428	chr15	45003746	T>A	43.0	453	B2M	SNV	M1K
L1236	chr12	57496662	C>T	88.7	8647	STAT6	SNV	D419N
L1236	chr12	57496668	T>A	88.9	8580	STAT6	SNV	N417Y
L591	chr5	149450132	T>C	46.2	492	CSF1R	SNV	H362R
