# Supplementary material for: Analysis of the mutational landscape of classic Hodgkin lymphoma identifies disease heterogeneity and potential therapeutic targets
Source: Oncotarget. 2017 Nov 30;8(67):111386–95. doi: 10.18632/oncotarget.22799 (PMC5762329; doi:10.18632/oncotarget.22799)
Supplement: Supplementary file 2 [file oncotarget-08-111386-s002.docx]

**Supplementary Table 1: Target selection design (Sure Select)**

| **BCR pathway** | | | **DLBCL Pathway** | **NFKB pathway** | | | | **STAT pathway** | | **TCR pathway** | | **HL pathway** | |
| --- | --- | --- | --- | --- | --- | --- | --- | --- | --- | --- | --- | --- | --- |
| AKT1 | JUN | PIK3CB | ADAM8 | AHR | GADD45B | NFKB1 | TNFAIP3 | BCL2L1 | IL6 | CBL | MAPK3 | TNFRSF5 | CENPF |
| AKT2 | KRAS | PIK3CD | AFMID | BAG4 | ICAM1 | NFKB2 | TNFAIP6 | IL10 | IL6R | CBLB | NCK1 | TNFSF5 | MAPRE1 |
| AKT3 | LPL | PIK3CG | ARID3A | BANK1 | ID2 | NFKBIA | TNFRSF10A | IL10RA | IL6ST | CBLC | NCK2 | CD80 | BUB3 |
| B2M | LYN | PIK3R1 | BCL6 | BATF | IGF1 | NFKBIB | TNFRSF10B | IL10RB | IL7 | CD247 | PAK1 | CD86 | NBS1 |
| BCL10 | MAP2K3 | PIK3R2 | BCOR | BCL2 | IKBKAP | NFKBIE | TNFRSF11A | IL11 | IL7R | CD28 | PAK2 | FUT4 | CCNH |
| BLNK | MAP2K4 | PIK3R3 | CD1A | BCL2A1 | IKBKB | NFKBIL1 | TNFRSF11B | IL11RA | IL9 | CD3D | PAK3 | FUT9 | CSE1L |
| BTK | MAP2K7 | PIK3R5 | CKAP4 | BCL3 | IKBKE | NFKBIL2 | TNFRSF12A | IL12A | IL9R | CD3E | PAK6 | FUT7 | NUMA1 |
| CALM1 | MAP3K13 | PIM2 | DGKG | BIRC2 | IKBKG | NGFR | TNFRSF13B | IL12B | IRF9 | CD3G | PAK7 | FUT6 | CDC2 |
| CALM2 | MAP3K3 | PIM3 | DUSP5 | BIRC3 | IL1A | PASK | TNFRSF13C | IL12RB1 | JAK1 | CD4 | PDCD1 | CASP8 | HMMR |
| CALM3 | MAP3K6 | PLCG2 | ENPP3 | BIRC4 | IL1B | PBEF1 | TNFRSF14 | IL12RB2 | JAK2 | CD8A | PDK1 | CASP3 | RSN |
| CARD11 | MAP4K4 | PRDM1 | ERCC6L | BIRC5 | IL1R1 | PECAM1 | TNFRSF17 | IL13 | JAK3 | CD8B | PLCG1 | CASP9 | RAMP |
| CD19 | MAPK8 | PRKCB | EZH2 | BIRC6 | IL32 | PLEK | TNFRSF18 | IL13RA1 | LIF | CDC42 | PPP3CA | BAX | CCNH |
| CD22 | MCL1 | PRKD1 | GSG2 | BIRC7 | IL8 | PRKCA | TNFRSF19 | IL13RA2 | LIFR | CDK4 | PPP3CB | BAK1 | CDK7 |
| CD27 | MS4A1 | PRKD3 | IGLL1 | BLK | IRAK1 | PRKCD | TNFRSF1A | IL15 | MPL | CSF2 | PPP3CC | BID | CCNA2 |
| CD38 | MyD88 | PTPN1 | irak3 | CCL2 | IRF1 | PRKCH | TNFRSF1B | IL15RA | MYC | CSK | PPP3R1 | BIK | CDC6 |
| CD5 | NAIP | PTPRC | LFNG | CCL22 | IRF3 | PRKCI | TNFRSF21 | IL19 | PIAS1 | CTLA4 | PPP3R2 | BCL2L11 | CCNE2 |
| CD70 | NFAM1 | RELT | LPP | CCL3 | ITGAL | PRKCZ | TNFRSF25 | IL2 | PIAS2 | DAPP1 | PRKCQ | MCL1 | CDC2 |
| CD79A | NFAT5 | RHOA | MME | CCL4 | ITGAM | PTPN3 | TNFRSF4 | IL20 | PIAS3 | DOK1 | PTEN | BCL2L10 | BCCIP |
| CD79B | NFATC1 | SMARCA4 | NCF4 | CCR4 | JUNB | REL | TNFRSF6B | IL20RA | PIAS4 | DOK2 | PTPN7 | BAD | CCNH |
| CD81 | NFATC2 | STK40 | PECR | CCR7 | KLF10 | RELA | TNFRSF8 | IL21 | PIM1 | ELK1 | RAC1 | CSF2RA | CDKN2C |
| CHP | NFATC2IP | SYK | PFTK1 | CD36 | LITAF | RELB | TNFRSF9 | IL21R | PTPN6 | FOS | RAF1 | CSF3 | H1F0 |
| CHUK | NFATC3 | TCL1A | PLTP | CD40 | LSP1 | RET | TNFSF10 | IL22 | SOCS1 | GRAP2 | RASA1 | CSF2RB | H2AFX |
| CR2 | NFATC4 | TCL1B | PSTPIP2 | CD40LG | LTA | RGS1 | TNFSF11 | IL22RA1 | SOCS2 | GRB2 | RASGRP1 | CSF3R | HIST1H3D |
| CYLD | NFKBIZ | TLR2 | PTK2 | CD44 | LTB | RIPK1 | TNFSF12 | IL22RA2 | SOCS3 | HRAS | SHC1 | CSF1R | DCK |
| DAPP1 | NRAS | TP53 | PTPRO | CD82 | LTBR | RIPK2 | TNFSF13 | IL23R | SOCS4 | ICOS | SOS1 | CSF2 | RRM2 |
| GSK3B | OASL | TTRAP | RAB7L1 | CD83 | MALT1 | RIPK3 | TNFSF13B | IL26 | SOCS5 | IFNG | SOS2 | CSF1 | TYMS |
| IL17D | PAG1 | VAV1 | RASL11A | CEP110 | MAP3K1 | RRAS2 | TNFSF14 | IL28A | SOCS6 | ITK | TCRA | CD274 | MLH1 |
| IL1RAP | PAK4 | VAV2 | RC3H1 | CFLAR | MAP3K14 | SDC4 | TNFSF15 | IL28B | STAM | LAT | TCRB | PDCD1LG2 | TOP2A |
| IL27RA | PELI1 | VAV3 | RRAGB | CRADD | MAP3K4 | SELL | TNFSF18 | IL28RA | STAM2 | LCK | TEC | DNAJA2 | GRB2 |
| IL4RA | PIK3C2B | VPREB1 | RRAS2 | CX3CL1 | MAP3K5 | SMAD7 | TNFSF4 | IL29 | STAT1 | LCP2 | UBASH3B | HSPA4 | MAPK9 |
| INPP5D | PIK3C3 | VPREB3 | S100A8 | CXCL1 | MAP3K7 | SMARCA2 | TNFSF8 | IL2RA | STAT2 | MAP2K1 | ZAP70 | HSP90AA1 | MAPK6 |
| IRF4 | PIK3CA | WSB2 | SERPINA9 | CXCL10 | MAP3K7IP1 | SOD2 | TNFSF9 | IL2RB | STAT3 | MAP4K1 | NOTCH1 | HSPA9B | SH2D1A |
| **MCL Pathway** | | | SH3BP5 | CXCL13 | MAP3K7IP2 | SPI1 | TRADD | IL2RG | STAT4 | **Others** | | AURKA | ALDH1A1 |
| ATM | CDKN2A | MDM2 | TOX | CXCL2 | MAP3K7IP3 | SPIB | TRAF1 | IL3 | STAT5A | RYBP | MEL18 | CENPE | ITGA4 |
| BMI1 | CEBPB | RB1 | TPD52 | CXCL9 | MAP3K8 | TANK | TRAF2 | IL3RA | STAT5B | RNF2 | GLI3 | MAD2L1 | LCP1 |
| CCND1 | CHEK1 | SUZ12 | ZBTB32 | CXCR4 | MAPK11 | TBK1 | TRAF3 | IL4 | STAT6 | RING1 |  | BUB1B | LGALS1 |
| CDK6 | CHEK2 |  | ZNF185 | CXCR7 | MAPK12 | TLR4 | TRAF4 | IL5 | TSLP |  |  |  |  |
|  |  |  | ZNF230 | FADD | MAPK13 | TNF | TRAF5 | IL5RA | TYK2 |  |  |  |  |
|  |  |  | KLHL6 | FAS | MAPK14 | TNFAIP1 | TRAF6 |  |  |  |  |  |  |
|  |  |  |  | FYN | MYB | TNFAIP2 |  |  |  |  |  |  |  |
