# Supplementary material for: Analysis of the mutational landscape of classic Hodgkin lymphoma identifies disease heterogeneity and potential therapeutic targets
Source: Oncotarget. 2017 Nov 30;8(67):111386–95. doi: 10.18632/oncotarget.22799 (PMC5762329; doi:10.18632/oncotarget.22799)
Supplement: Supplementary file 3 [file oncotarget-08-111386-s003.docx]

**Supplementary Table 2: Ampliseq custom panel**

|  |  | **COVERAGE STATUS** | | | | **DESIGN CRITERIA** | | | |
| --- | --- | --- | --- | --- | --- | --- | --- | --- | --- |
| **GENE** | **SEQUENCING** | **AMPLICONS** | **TOTAL BASES** | **COVERED BASES** | **OVERALL COVERAGE (%)** | **cHL** | **PREVIOUS SEQUENCING** | **DLBCL** | **BIOLOGICAL RELEVANCE** |
| **B2M** | **COMPLETE GENE** | 6 | **393** | **393** | **100** | **X** | **X** | **X** |  |
| **CARD11** |  | 49 | **3729** | **3674** | **98,52** |  | **X** |  |  |
| **CASP8** |  | 23 | **2263** | **2232** | **98,63** |  | **X** |  |  |
| **CSF1R** |  | 42 | **3150** | **3150** | **100** |  | **X** |  |  |
| **BCL10** |  | 11 | **735** | **704** | **95,78** |  | **X** |  |  |
| **NFKBIA** |  | 14 | **1020** | **970** | **95,09** | **X** | **X** |  |  |
| **STAT6** |  | 37 | **2795** | **2795** | **100** |  | **X** |  |  |
| **BTK** |  | 28 | **2178** | **2153** | **98,85** |  | **X** |  |  |
| **IL32** |  | 10 | **850** | **760** | **89,41** |  | **X** |  |  |
| **MYB** |  | 34 | **2787** | **2721** | **97,63** |  |  |  | **X** |
| **SH3BP5** |  | 16 | **1502** | **1296** | **86,28** |  | **X** |  |  |
| **ABL1** | **REGION** | 8 | **3393** | **431** | **12,7** |  | **X** |  |  |
| **ADAM8** |  | 4 | **2229** | **178** | **8** |  | **X** |  |  |
| **CD19** |  | 3 | **1674** | **144** | **8,6** |  | **X** |  |  |
| **CD38** |  | 2 | **903** | **88** | **9,7** |  | **X** |  |  |
| **NFKB2** |  | 5 | **2700** | **238** | **8,8** |  | **X** |  |  |
| **PIK3CD** |  | 5 | **3135** | **205** | **6,5** |  | **X** |  |  |
| **RET** |  | 8 | **3219** | **391** | **12,14** |  | **X** |  |  |
| **TNFRSF14** |  | 1 | **852** | **58** | **6,8** |  | **X** |  |  |
| **CSF2RB** |  | 4 | **2694** | **164** | **6** | **X** | **X** |  |  |
| **FAS** |  | 1 | **7536** | **41** | **0,5** | **X** |  |  | **X** |
| **LCP1** |  | 1 | **1866** | **41** | **2,2** |  |  |  | **X** |
| **LTB** |  | 2 | **234** | **110** | **47** |  | **X** |  |  |
| **MAPK3** |  | 2 | **1074** | **82** | **7,6** |  | **X** |  |  |
| **MDM2** |  | 2 | **966** | **82** | **8,4** |  |  |  | **X** |
| **NUMA1** |  | 5 | **6306** | **205** | **3,2** |  | **X** |  |  |
| **PLCG2** |  | 3 | **3798** | **131** | **3,4** |  | **X** |  |  |
| **SMARCA4** |  | 7 | **4842** | **322** | **6,6** |  | **X** |  |  |
| **NOTCH1** |  | 4 | **7668** | **269** | **3,5** |  | **X** | **X** |  |
| **CREBBP** |  | 3 | **7215** | **252** | **3,4** |  |  | **X** |  |
| **EP300** |  | 8 | **7245** | **460** | **6,3** |  |  | **X** |  |
| **STAT3** |  | 1 | **2169** | **103** | **4,7** |  |  | **X** |  |
| **MYC** |  | 2 | **1365** | **82** | **6** |  |  | **X** |  |
| **EZH2** |  | 1 | **2214** | **25** | **1,1** |  |  | **X** |  |
| **MYD88** |  | 1 | **480** | **44** | **9,1** |  |  | **X** |  |
