# Supplementary material for: Analysis of the mutational landscape of classic Hodgkin lymphoma identifies disease heterogeneity and potential therapeutic targets
Source: Oncotarget. 2017 Nov 30;8(67):111386–95. doi: 10.18632/oncotarget.22799 (PMC5762329; doi:10.18632/oncotarget.22799)
Supplement: Supplementary file 4 [file oncotarget-08-111386-s004.docx]

**Supplementary Table 3: Selected genes, primers and PCR conditions for Sanger sequencing of DNA extracted from cell lines**

| **Gene** | **Forward Primer** | **Reverse Primer** | **Genomic region (hg19) bp** | **Annealing Temperature** | **Amplicon length (bp)** |
| --- | --- | --- | --- | --- | --- |
| STAT6 | CCCCTGTTCCCTCCAACT | AACCCCTGTCCTCACCCT | 57496578-57496728 | 60 | 151 |
| CSF2RB | AGCACCCACTGTCTCCTGA | AAACCTCTGGGCTCCACTTG | 37325659-37325831 | 60 | 173 |
| CARD11 | CGGTAGGTGCTCCGGAGA | CAGGGCCTGACTGATTGATAAATTCA | 2956899-2957051 | 60 | 153 |
| CARD11 | CACCCTTGGGGTATTTCAGA | CAGGCCCTCACCTGGATG | 2979372-2979581 | 60 | 210 |
| NFKBIA | CACTCTCTGGCAGCATCTGAA | AAAATCCTGACCTGGTGTCACTC | 35871656-35871823 | 60 | 168 |
| B2M | GGCTGGGCACGCGTTTAATATA | GGGTAGGAGAGACTCACGCT | 45003669-45003828 | 60 | 160 |
