# Supplementary material for: Analysis of the mutational landscape of classic Hodgkin lymphoma identifies disease heterogeneity and potential therapeutic targets
Source: Oncotarget. 2017 Nov 30;8(67):111386–95. doi: 10.18632/oncotarget.22799 (PMC5762329; doi:10.18632/oncotarget.22799)
Supplement: Supplementary file 5 [file oncotarget-08-111386-s005.docx]

**Supplementary Table S4: SNV summary**

| **Gene ID** | **Sample** | **Chromosome** | **Position** | **Base**  **change** | **Frequency** | **Coverage** | **Type** | **Aminoacid change** |
| --- | --- | --- | --- | --- | --- | --- | --- | --- |
| EP300 | 46 | chr22 | 41566451 | G>A | 3.1 | 362 | SNV | G1443E |
| EP300 | 67 | chr22 | 41566502 | C>T | 4.7 | 1766 | SNV | P1460L |
| EP300 | 30 | chr22 | 41566552 | G>A | 3.2 | 284 | SNV | E1477K |
| EP300 | 42 | chr22 | 41566567 | G>A | 3.29 | 547 | SNV | D1482N |
| EP300 | 29 | chr22 | 41569639 | G>A | 3.2 | 791 | SNV | D1544N |
| EP300 | 97 | chr22 | 41572281 | C>T | 4.9 | 487 | SNV | P1604S |
| EP300 | 16 | chr22 | 41572356 | G>A | 4.02 | 1070 | SNV | A1629T |
| EP300 | 67 | chr22 | 41572357 | C>T | 5.2 | 559 | SNV | A1629V |
| BTK | 18 | chrX | 100609636 | G>A | 3 | 199 | SNV | S538F |
| BTK | 67 | chrX | 100613298 | C>T | 5.2 | 517 | SNV | G368R |
| BTK | 18 | chrX | 100613683 | C>T | 3.3 | 269 | SNV | G299E |
| BTK | 85 | chrX | 100625027 | G>A | 3.3 | 424 | SNV | T117I |
| BTK | 46 | chrX | 100629565 | C>T | 4.1 | 241 | SNV | V67M |
| BTK | 81 | chrX | 100629576 | C>T | 3.5 | 742 | SNV | C63Y |
| BTK | 48 | chrX | 100629615 | C>T | 3.18 | 440 | SNV | G50D |
| CSF2RB | 83 | chr22 | 37325765 | G>A | 8.3 | 103 | SNV | V212I |
| CSF2RB | 81 | chr22 | 37325765 | G>A | 4.1 | 369 | SNV | V212I |
| CSF2RB | 82 | chr22 | 37325765 | G>A | 11.5 | 103 | SNV | V212I |
| CSF2RB | 79 | chr22 | 37325765 | G>A | 40 | 153 | SNV | V212I |
| CSF2RB | 29 | chr22 | 37334299 | C>T | 4.4 | 720 | SNV | Q823* |
| CSF2RB | 46 | chr22 | 37334369 | C>T | 4.2 | 311 | SNV | S846F |
| CSF2RB | 85 | chr22 | 37334375 | C>T | 4.4 | 159 | SNV | P848L |
| STAT6 | 29 | chr12 | 57492684 | C>T | 3.3 | 996 | SNV | D653N |
| STAT6 | 42 | chr12 | 57493579 | G>A | 3.02 | 1028 | SNV | T572I |
| STAT6 | 67 | chr12 | 57496194 | C>T | 4 | 2851 | SNV | G464E |
| STAT6 | 83 | chr12 | 57496654 | A>T | 6.9 | 853 | SNV | N421K |
| STAT6 | 13 | chr12 | 57496661 | T>A | 4 | 3648 | SNV | D419V |
| STAT6 | 34 | chr12 | 57496662 | C>G | 4.7 | 1710 | SNV | D419H |
| CARD11 | 28 | chr7 | 2953029 | A>C | 5.7 | 212 | SNV | C971G |
| CARD11 | 81 | chr7 | 2959028 | C>T | 3.2 | 157 | SNV | G830S |
| CARD11 | 67 | chr7 | 2962959 | C>T | 4.2 | 526 | SNV | R650Q |
| CARD11 | 42 | chr7 | 2969639 | G>A | 4.08 | 515 | SNV | S547F |
| CSF1R | 16 | chr5 | 149433711 | G>A | 6.26 | 655 | SNV | S947F |
| CSF1R | 29 | chr5 | 149435793 | C>T | 3.9 | 840 | SNV | V811I |
| CSF1R | 30 | chr5 | 149440433 | G>A | 5.1 | 198 | SNV | T654I |
| CSF1R | 82 | chr5 | 149460452 | G>A | 4.1 | 122 | SNV | S62F |
| MYB | 29 | chr6 | 135509006 | G>A | 4.9 | 103 | SNV | G59E |
| MYB | 97 | chr6 | 135511360 | G>A | 3.7 | 163 | SNV | W134* |
| MYB | 16 | chr6 | 135515515 | C>T | 7.80 | 487 | SNV | P289S |
| MYB | 35 | chr6 | 135518109 | C>T | 3.21 | 1496 | SNV | S405L |
| ABL1 | 67 | chr9 | 133738305 | G>A | 3.6 | 638 | SNV | W254* |
| ABL1 | 81 | chr9 | 133738340 | A>G | 7.6 | 1836 | SNV | K266R |
| ABL1 | 82 | chr9 | 133738340 | A>G | 7.4 | 236 | SNV | K266R |
| B2M | 15 | chr15 | 45003746 | T>C | 2.9 | 2000 | SNV | M1T |
| B2M | 23 | chr15 | 45007811 | C>G | 3.7 | 349 | SNV | Y86* |
| BCL10 | 48 | chr1 | 85733585 | AATT>- | 18.7 | 1919 | Deletion | FRAMSHIP |
| BCL10 | 29 | chr1 | 85736499 | C>T | 3.7 | 216 | SNV | E50K |
| CD19 | 29 | chr16 | 28950202 | C>T | 3.6 | 1092 | SNV | S531F |
| CD19 | 29 | chr16 | 28950268 | C>T | 3 | 1059 | SNV | T553I |
| NFKBIA | 35 | chr14 | 35871981 | G>A | 3.57 | 672 | SNV | A211V |
| NFKBIA | 96 | chr14 | 35872990 | G>A | 5.4 | 2844 | SNV | A81V |
| CASP8 | 79 | chr2 | 202149992 | C>T | 9 | 1102 | SNV | T478I |
| CD38 | 97 | chr4 | 15826622 | G>A | 6.6 | 136 | SNV | G161D |
| CREBBP | 70 | chr16 | 3781255 | C>T | 3.3 | 241 | SNV | V1734I |
| CSF2 | 67 | chr5 | 131409526 | C>T | 3.7 | 406 | SNV | Q4* |
| FAS | 29 | chr10 | 90774035 | G>A | 3.8 | 132 | SNV | R306H |
| LCP1 | 16 | chr13 | 46704953 | C>T | 3.96 | 1539 | SNV | A583T |
| MYC | 81 | chr8 | 128750632 | C>T | 4.7 | 105 | SNV | P57S |
| NOTCH1 | 24 | chr9 | 139404248 | C>CG | 4.18 | 1291 | Insertion | FRAMESHIP |
| PIK3CD | 85 | chr1 | 9775710 | G>A | 4.7 | 430 | SNV | E85K |
| RET | 67 | chr10 | 43620335 | C>T | 36.1 | 551 | SNV | R982C |
| SH3BP5 | 35 | chr3 | 15297639 | C>T | 4.38 | 776 | SNV | G441E |
| SMARCA4 | 85 | chr19 | 11134230 | C>T | 3 | 266 | SNV | R1030W |
